# Hydrogel-Based Biointerfaces: Recent Advances, Challenges, and Future Directions in Human–Machine Integration

**DOI:** 10.3390/gels11040232

**Published:** 2025-03-23

**Authors:** Aziz Ullah, Do Youn Kim, Sung In Lim, Hyo-Ryoung Lim

**Affiliations:** 1 Major of Human Bioconvergence, Division of Smart Healthcare, College of Information Technology and Convergence, Pukyong National University, Busan 48513, Republic of Korea; azizullah1@pknu.ac.kr (A.U.); doyoun6789@gmail.com (D.Y.K.); 2 Department of Chemical Engineering, Pukyong National University, Busan 48513, Republic of Korea

**Keywords:** human–machine interfacing, hydrogels, biocompatible materials, wearable electronics, conductive hydrogels, neural interfaces

## Abstract

Human–machine interfacing (HMI) has emerged as a critical technology in healthcare, robotics, and wearable electronics, with hydrogels offering unique advantages as multifunctional materials that seamlessly connect biological systems with electronic devices. This review provides a detailed examination of recent advancements in hydrogel design, focusing on their properties and potential applications in HMI. We explore the key characteristics such as biocompatibility, mechanical flexibility, and responsiveness, which are essential for effective and long-term integration with biological tissues. Additionally, we highlight innovations in conductive hydrogels, hybrid and composite materials, and fabrication techniques such as 3D/4D printing, which allow for the customization of hydrogel properties to meet the demands of specific HMI applications. Further, we discuss the diverse classes of polymers that contribute to hydrogel conductivity, including conducting, natural, synthetic, and hybrid polymers, emphasizing their role in enhancing electrical performance and mechanical adaptability. In addition to material design, we examine the regulatory landscape governing hydrogel-based biointerfaces for HMI applications, addressing the key considerations for clinical translation and commercialization. An analysis of the patent landscape provides insights into emerging trends and innovations shaping the future of hydrogel technologies in human–machine interactions. The review also covers a range of applications, including wearable electronics, neural interfaces, soft robotics, and haptic systems, where hydrogels play a transformative role in enhancing human–machine interactions. Thereafter, the review addresses the challenges hydrogels face in HMI applications, including issues related to stability, biocompatibility, and scalability, while offering future perspectives on the continued evolution of hydrogel-based systems for HMI technologies.

## 1. Introduction

Over the past century, interdisciplinary collaborations in medicine, biology, and biomedical engineering have advanced our understanding of the human body [1]. The development of advanced machine systems—including computers, mobile devices, sensors, actuators, and robots—has progressed from science fiction to everyday reality [2]. However, artificial interfaces mediating human–machine interactions remain underdeveloped, often resulting in short-lived and inefficient communication.

Emerging fields such as brain–machine interfaces (BMIs), neuroprosthetics, clinical medical equipment, medical implants, wearable and ingestible devices, and virtual/augmented reality highlight the limitations of the current interface technologies [3]. Achieving seamless, biocompatible human–machine connectivity could revolutionize health care and technology, yet persistent challenges remain.

Conventional BMI probes such as Michigan and Utah arrays often trigger foreign body reactions, causing gliosis and scar tissue formation [4,5]. Likewise, immune responses compromise the long-term efficacy of implantable glucose sensors and insulin pumps, leading to sensor–tissue detachment [6,7]. A fundamental issue with the current interface devices is the materials themselves. Traditional components—metals, silicon, glass, ceramics, and plastics—are rigid and abiotic, which makes them incompatible with the soft, flexible, and dynamic nature of biological tissues. Recent research has focused on modifying these materials to create flexible and stretchable structures that can better interface with biological tissues [8,9]. Despite progress, these materials still face significant challenges. Preoperative attachment or suturing of conventional materials often leads to nonconformal insertion, unstable bonding, tissue puncture, and scarring [10,11]. In particular, metallic electrodes, with their inherent rigidity, exhibit limited interfacial capacitance and charge injection capacity, resulting in reduced effectiveness in electrical monitoring or stimulation. Moreover, these materials are perceived as foreign by biological tissues, triggering biofouling, fibrotic encapsulation, and other adverse responses that restrict proper integration [12,13].

**Figure 1:**
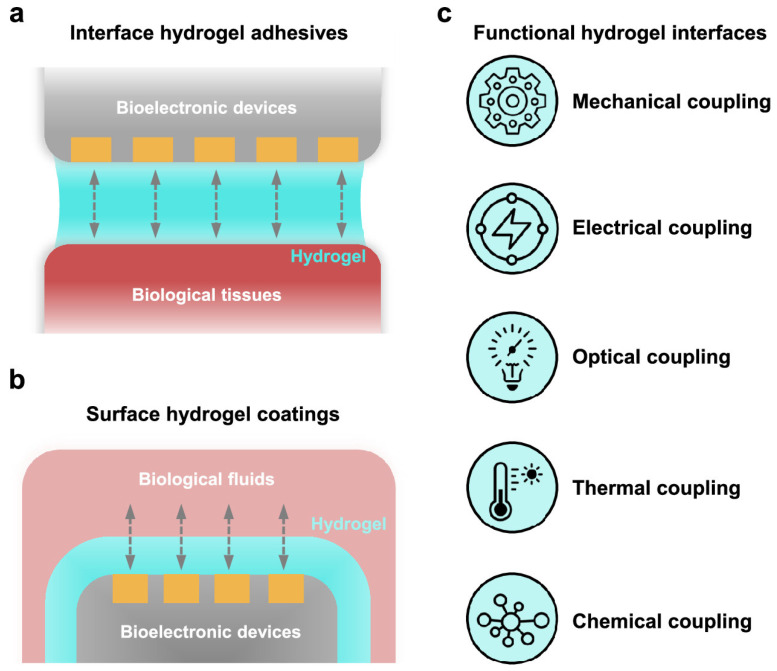
Schematic illustration of hydrogel-based biointerfaces for advanced bioelectronic applications. (**a**) Illustration of a hydrogel interface enabling seamless interaction between biological tissues and electronic systems. (**b**) Representation of a hydrogel coating applied to a device to enhance biocompatibility and functional performance. (**c**) Summary of the key hydrogel attributes—mechanical adaptability, conductivity, transparency, thermal regulation, and chemical responsiveness—enhancing human–machine interactions. Reproduced with permission from Ref. [34].

Hydrogels have emerged as promising materials for HMIs due to their tissue-mimicking properties, tunability, and biocompatibility [14,15]. Hydrogel-based interfaces have advanced significantly in both research and commercial applications. Ultrasound coupling hydrogels are now standard in medical imaging and therapy [16]. Skin-adhesive hydrogels are widely used in bioelectronics, including electrocardiography, electromyography, electroencephalography, and transcutaneous electrical nerve stimulation [17,18]. Despite these advancements, HMI remains rudimentary, especially in BMIs, neuroprosthetics, and wearables. Challenges stem from conventional materials such as metals, silicon, and plastics, which are rigid, abiotic, and nonconformal, leading to biofouling, fibrosis, and poor long-term integration [13,19]. Hydrogels offer a promising alternative with their biocompatibility and tissue-mimicking properties [20,21]. Figure 1 illustrates hydrogel-based biointerfaces designed for seamless biological–electronic integration, enhancing bioelectronic applications such as wearable sensors and medical devices [22,23]. Hydrogels also show potential in wearable technologies, including sweat sensors [24], contact lenses [25], and wound dressings [26]. Their tunability and biodegradability make them suitable for ingestible sensors, while their biocompatibility reduces biofouling, improving implant longevity [27,28,29,30].

Despite their promise, hydrogel-based HMIs remain underexplored. While the existing reviews focus on tissue engineering [31], drug delivery [32], and soft robotics [33], they often overlook their role in HMIs. This review highlights recent advances, design principles, and applications of hydrogels in wearable, neural, and implantable interfaces. By addressing challenges in biocompatibility, flexibility, and integration, it aims to advance next-generation hydrogel-based HMIs.

## 2. Critical Attributes of Hydrogels for HMI Integration

### 2.1. Biocompatibility

For wearable electronic devices that make direct contact with the skin, ensuring biocompatibility is essential. Biocompatible antifreeze hydrogels offer dual advantages by maintaining operational stability at low temperatures and providing robust protection to the skin. These hydrogels are designed to function effectively in various environmental conditions, making them suitable for applications such as wearable sensors and electronic skin [35]. Biocompatibility testing of hydrogels is essential to ensure their safety and effectiveness in medical applications. This evaluation typically involves two primary methods: in vitro cell culture assessments and in vivo animal studies. In vitro testing involves culturing cells within the hydrogel matrix under controlled laboratory conditions. This approach allows for the assessment of cell proliferation, morphology, and potential toxicity, providing insights into the hydrogel’s compatibility with biological tissues. Live/dead assays, MTT (3-(4,5-dimethylthiazol-2-yl)-2,5-diphenyltetrazolium bromide) assays, and immunohistochemical staining are commonly used techniques for testing hydrogels in biocompatibility studies [36]. In vivo studies involve the administration of the hydrogel to live animal models to assess tissue recovery and the organism’s physiological response to material exposure. These studies provide critical insights into the material’s biocompatibility, including its interaction with surrounding tissues, potential immune responses, and overall safety in a living system. Depending on the intended application and relevant regulatory standards, selecting the appropriate biocompatibility testing method is essential to ensure the material’s safety and suitability for its specific use. These methods must align with the potential clinical or environmental conditions the hydrogel will encounter to provide reliable data on its biological interactions. For example, Wang et al. (2019) performed frostbite experiments on a prepared antifreeze organo-hydrogel (PEDOT:SL-PAA). The authors examined animal skin by applying an organo-hydrogel (PEDOT:SL-PAA) and determined that the rats covered with this material sustained no tissue damage [37]. Through histological analysis, researchers have confirmed that the structure of organo-hydrogels effectively shields both the epidermal skin layers and collagen fibers. Laboratory evaluations of the organo-hydrogel’s toxicity to human skin revealed minimal cytotoxic effects, supporting its suitability for direct skin contact in wearable medical devices. Researchers conducted frostbite experiments on rat skin with M-PVA hydrogel coverage, which proved its ability to guarantee a fully protected epidermis and demonstrated outstanding antifreeze hydrogel performance in skin protection applications [38]. Additionally, anti-icing hydrogels made from antifreeze proteins sourced from deep-sea fish have demonstrated outstanding biocompatibility. For instance, Xu et al. (2020) [39] and Wang et al. (2022) [40] conducted biocompatibility assessments in their respective studies, which included prolonged skin application and cytotoxicity tests. The findings from both studies confirmed that these antifreeze, protein-based hydrogels exhibit excellent biocompatibility and do not cause skin damage upon removal.

### 2.2. Electrical Conductivity

Electronic devices intended for wearable HMI applications must possess electrical conductivity as their core functional attribute. Incorporating inorganic salts into hydrogels represents a basic yet affordable way to improve ionic conductivity and expand the application range for antifreeze hydrogels [41,42]. In their 2021 study, Di et al. developed an ion-conductive hydrogel by cross-linking poly(acrylamide-N-acryloyl-2-glycine) with lithium chloride and sodium chloride, achieving an electrical conductivity of 1.35 S·m^−1^ at room temperature. This hydrogel demonstrated enhanced flexibility and sensor detection capabilities across a broad temperature range, offering valuable insights for advancing freeze-resistant hydrogels [43]. Wang et al. (2023) developed a zwitterionic hydrogel by copolymerizing sulfobetaine methacrylate and acrylamide, resulting in a material with high ionic conductivity and antifreeze properties. The hydrogel maintained an impressive ionic conductivity of 12.6 mS/cm even at −40 °C, demonstrating its potential for low-temperature applications [44]. Recent studies have demonstrated that hydrogels can function effectively as circuit conductors in light-emitting diode (LED) applications at low temperatures, maintaining light levels comparable to those at room temperature. For instance, a hydrogel composed of polyvinyl alcohol (PVA) and carboxymethyl cellulose (CMC) infused with Zn(CF_3_SO_3_)_2_ exhibited stable conductivity at temperatures as low as −20 °C, enabling consistent LED illumination [45]. Conventional ionically conductive hydrogels often face challenges such as weak mechanical properties and limited tensile behavior, which can hinder their practical applications. To address these issues, incorporating conductive fillers into polymer networks has emerged as an effective strategy to enhance both mechanical strength and electrical conductivity. For instance, adding carbon-based nanomaterials such as carbon nanotubes (CNTs) or graphene can significantly improve the mechanical robustness and conductive properties of hydrogels. However, it is important to note that while conductive fillers can significantly enhance electrical conductivity, they often lead to reduced stretchability and inferior mechanical properties if not properly integrated [46]. Therefore, careful design and optimization are essential to balance conductivity and mechanical performance in antifreeze hydrogels. For example, Wang et al. developed an organic antifreeze hydrogel by incorporating poly(3,4-ethylenedioxythiophene): sulfonated lignin (PEDOT:SL) fillers into a polyacrylic acid (PAA) network using a glycerol/water binary solvent. This hydrogel exhibited excellent antifreeze properties and water retention capabilities. Notably, the strain sensitivity of the hydrogel was significantly enhanced after replacing the solvent, enabling precise monitoring of biological signals such as electrocardiograms (ECG) and electromyograms (EMG) [37].

### 2.3. Optical Clarity

The ability of wearable sensors to maintain transparency opens up a wide range of applications, such as electronic skins and touchscreens for human–machine interaction. PVA-based hydrogels, known for their exceptional optical properties, are particularly well-suited for use in wearable electronics. These hydrogels often achieve transparency by utilizing PVA and PAAm as the primary materials. For example, a dual-network antifreeze hydrogel composed of polyvinyl alcohol and polyacrylamide (PVA/PAAm) and prepared using a mixture of ethylene glycol (EG) and water as solvents has shown remarkable optical performance, with an 82% transmission efficiency at temperatures as low as −50 °C [47]. Additionally, a multifunctional antifreeze hydrogel constructed from a cross-linked copolymer of poly(2-(methacryloyloxy) ethyl dimethyl-(3-sulfopropyl) ammonium hydroxide-co-acrylamide) P(SBMA-co-AAm) exhibited impressive optical clarity, achieving an 80.6% transparency within 24 h of preparation. Even after one month, this hydrogel maintained a high degree of transparency, ca. 75.2%, demonstrating its exceptional stability [48]. These transparent, frost-resistant hydrogels offer significant potential for flexible touchscreens and other wearable electronics that need to operate effectively in cold environments.

### 2.4. Intrinsic Healing Behavior

Self-healing processes commonly observed in living organisms, such as the natural repair of human skin after injury, play a crucial role in extending the lifespan, stability, and functionality of materials and devices, especially in extreme conditions. This unique mechanical property is typically realized through mechanisms such as hydrogen bonding, metal–ligand interactions, dynamic covalent bonding, reversible ionic cross-linking, supramolecular interactions, or self-association [49]. New materials research has become focused on developing applications that employ self-healing properties. Using recently developed conductive hydrogel sensors, Wang et al. (2018) were able to monitor pulses and tiny strains with high sensitivity through self-healing units that reach a recovery efficiency of 97% while maintaining freeze-resistant properties [50]. The existing hydrogel bonds between borate esters and hydrogens enable contact with ions to trigger hydrogel self-healing when the cut hydrogel makes contact again. After healing, the hydrogel demonstrates a 1300% extension capability while maintaining structural integrity and supporting weights of up to 200 g. Multiple cycles of stimulation minimally affected the refurbished hydrogels’ electrical resistance value, showing outstanding product durability and structural stability. Liu et al. (2020) used freeze-resistant polyionic hydrogels (PIL hydrogels) to make artificial skin with three operational modes of self-healing ability as well as hyperstretchability and high electrical conductivity [51]. Self-healing excellence in these materials is formed through hydrogen bonds that generate reversible cross-links between acrylamide ionic contacts and amphoteric ILs. The hydrogel sustained healing throughout a 6 h period when placed in a 20-degrees-Celsius solution following incision. Gong et al. (2022) synthesized a multifunctional conductive hydrogel named CEH using a LiCl/EG/water freezing solution [52]. The self-healing hydrogel demonstrates superior resistance to freezing at low temperatures along with high ionic transport efficiency. The reassembled hydrogel achieves critical self-healing properties while recuperating at normal room temperature for two hours. This hydrogel material demonstrates an excellent self-healing capability, reaching 92.28% tensile and 92.78% compressive efficiency. The CEH demonstrates excellent self-healing attributes at temperatures up to 35 °C. Moreover, Liao et al. (2019) developed self-healing hydrogels through supramolecular interactions between EG and PVA and MXene and through dynamic hydroxyl–PVA tetrahydroxyborate cross-links that yielded optimal healing abilities [53].

### 2.5. Adhesion

Adhesion plays a crucial role in enhancing the sensing performance of flexible electronic devices. By improving the adhesion properties of hydrogels, the gaps between the hydrogel and the matrix are minimized, which optimizes the conversion of collected signals into electrical signals and enhances the accuracy of sensor monitoring. Incorporating amphoteric ions into hydrogels enhances their self-adhesive properties, improving sensor performance in flexible electronic systems. A study investigating an amphiphilic composite hydrogel found exceptional antifreeze properties and significant moisturizing benefits. Hexapolar hydrogels form noncovalent bonds that effectively bind amphoteric polymer chains to substrates, demonstrating high paper-binding capacity. Laboratory tests evaluating the composite hydrogel’s adhesion strength to various substrates at 30 °C showed only minimal enhancement compared to its performance at room temperature. The study provided a foundational understanding for developing flexible electronic devices that can function effectively in cold-temperature conditions [54].

Antifreeze proteins (AFP) within protein molecular chains enhance the adhesion properties of hydrogels as these proteins contain various functional groups, such as amino acids and carboxyl groups. These functional groups contribute to the superior adhesive characteristics and antifreeze capabilities of the hydrogel, enabling it to perform effectively under low-temperature conditions. A reported double-network antifreeze hydrogel sensor exhibited exceptional biocompatibility and strong adhesion capabilities to precisely detect human movements and minute physiological signals [39]. Zhao et al. (2021) explored the development of antifreeze hydrogels with exceptional adhesive and strain capabilities, enhanced by the incorporation of inorganic salts. By treating hydrogel filaments with a calcium chloride solution, remarkable improvements in electrical conductivity, ice protection, and auto-adhesion were observed. This treatment resulted in hydrogels that exhibited strong adhesive properties across various substrates, surpassing the peeling strength of medical bandages when applied to pig skin. Notably, these hydrogels maintained structural integrity even under a 200 g load, showcasing their resilience. The study highlights the promising manufacturing processes and properties of these hydrogels, which have significant potential for applications in bioelectronics and wearable electronics, where both flexibility and adhesion are crucial [55].

## 3. Recent Innovations in Hydrogel Design

**Figure 2:**
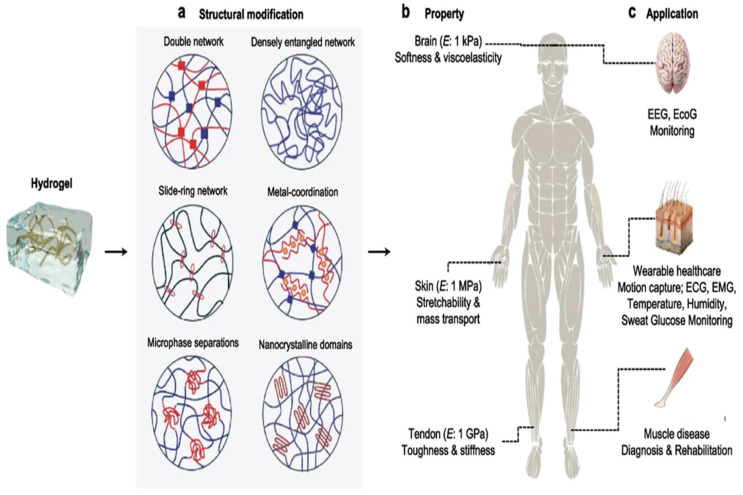
Approaches for modifying hydrogel properties and their use in bioelectronics. (**a**) Strategies for designing the network structure of hydrogels. (**b**) Mechanical characteristics of hydrogels that align with the properties of various human tissues. (**c**) Bioelectronic applications of hydrogels with customized properties, including electromyography, electroencephalography, electrocardiography, and electrocorticography. Reproduced from Ref. [61] under the Creative Commons Attribution 4.0 International License.

Recent innovations in hydrogel design have focused on enhancing their versatility and functionality across various applications, particularly in biomedical fields such as drug delivery, tissue engineering, and HMI. Researchers have developed hybrid hydrogels that combine natural and synthetic polymers to optimize mechanical properties and biocompatibility [56,57,58,59]. The integration of nanomaterials, such as graphene, carbon nanotubes, and MXenes, into hydrogel matrices has significantly improved their electrical conductivity, mechanical strength, and responsiveness to external stimuli, opening new possibilities for soft robotics and neural interfaces. Advances in 3D and 4D printing technologies have enabled the customization of hydrogel structures, allowing for the creation of dynamic and shape-morphing materials that can adapt to environmental changes. Additionally, microfluidic and electrospinning techniques have been employed to fabricate microstructured hydrogels with precise control over their morphology, advancing their use in bioelectronics and smart devices [60]. These innovations continue to push the boundaries of hydrogel functionality, offering exciting opportunities for their integration into a wide range of medical and technological applications. As shown in Figure 2, various strategies can be employed to tune hydrogel properties for the intended bioelectronic applications.

### 3.1. Conductive Hydrogels

Conductive hydrogels have emerged as a transformative material for HMI systems, offering a unique combination of electrical conductivity, mechanical flexibility, and biocompatibility. Their importance is particularly evident in applications such as neural interfaces, biosensors, and wearable electronics, where traditional rigid materials often fail due to poor long-term stability, mechanical mismatch with biological tissues, and immune response challenges [62]. In neural interfaces, such as BMIs and neuroprosthetics, conductive hydrogels provide a soft, conformal interface that reduces inflammatory responses while enhancing electrical signal transmission [29]. Unlike conventional metal electrodes, which can cause gliosis and scar formation, conductive hydrogels exhibit high ionic and electronic conductivity, ensuring stable signal recording and stimulation over extended periods [63]. Their self-healing and flexible nature further improve the durability of implanted electrodes, making them ideal for chronic applications. For example, a study demonstrated the use of a PEDOT: poly(styrene sulfonate) (PSS)-based hydrogel for neural interfacing, which exhibited excellent ionic and electronic conductivity, reduced impedance, and enhanced signal fidelity in chronic neural recordings [64]. The hydrogel also showed reduced inflammatory responses compared to traditional metal electrodes, highlighting its potential for long-term stability in neural applications.

In biosensors, conductive hydrogels enhance performance by facilitating efficient electron transfer, leading to increased sensitivity and rapid response times [65]. Their biocompatible matrix supports enzyme immobilization and bioelectrode functionalization, making them ideal for continuous monitoring of biomarkers such as glucose, lactate, cortisol, and neurotransmitters [66]. For instance, Pan et al. (2024) developed a self-healing, stretchable hydrogel incorporating MXene nanosheets for glucose sensing [67]. The material’s mechanical robustness ensured long-term functionality, making it a promising candidate for wearable biosensors [68]. Similarly, lactate biosensors for sports and medical applications utilize conductive hydrogels to ensure stable and reproducible electrochemical readings. Another notable example is provided by the hybrid hydrogel system composed of CNTs and silk fibroin for epidermal electrodes in ECG monitoring [69]. Similarly, other composite hydrogels have exhibited excellent stretchability, biocompatibility, and low impedance, outperforming traditional gel-based electrodes in signal stability and comfort [70]. These advancements highlight the potential of conductive hydrogels in improving the accuracy and reliability of biosensing technologies.

Wearable electronics, including electronic skins, motion sensors, and health-monitoring patches, also benefit from conductive hydrogels due to their stretchable and self-healing properties. These materials provide stable electrical networks that can withstand mechanical deformations, ensuring consistent signal collection in bioelectronic devices [71]. By offering ionically conductive pathways that mimic biological tissues, conductive hydrogels improve electrochemical sensing and actuation, making them integral to next-generation flexible and wearable electronics. For example, in applications such as epidermal electrodes for ECG and EMG, conductive hydrogels provide superior comfort, adherence, and signal fidelity compared to traditional gel-based electrodes. A recent study demonstrated the use of a liquid metal (LM)-doped PVA hydrogel for wearable strain sensors. The hydrogel exhibited high electrical conductivity and toughness through self-sintering LM microdroplets, making it suitable for motion tracking and health monitoring [72].

**Figure 3:**
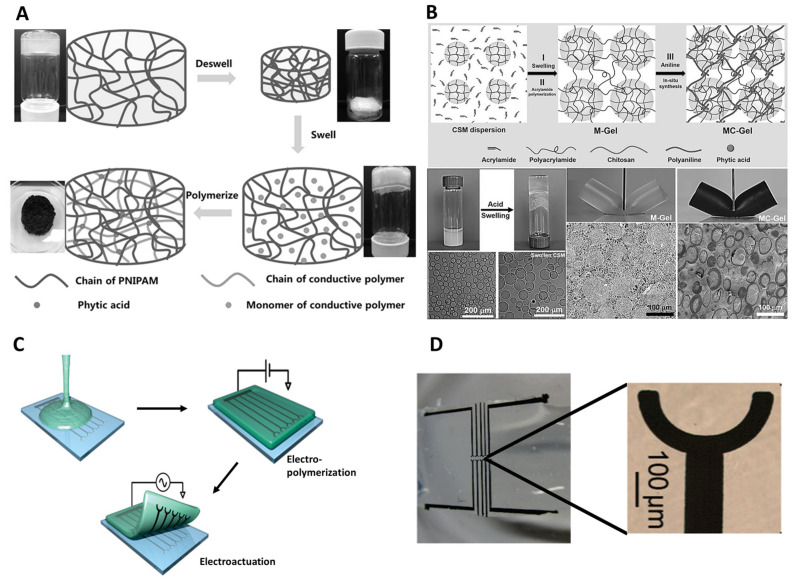
Fabrication of conductive polymer hydrogels. (**A**) Synthesis of PNIPAM-conductive polymer hybrid hydrogels [83] (Copyright 2015, Wiley-VCH). (**B**) In situ polymerization of PANI/PAAm within swollen chitosan microspheres [84] (Copyright 2016, Wiley-VCH). (**C**) Electro-polymerization-based fabrication of conductive polymer/hydrogel electrodes [85] (Copyright 2010, American Chemical Society). (**D**) PEDOT microelectrode array on a gel sheet [85] (Copyright 2010, American Chemical Society).

To enhance the conductivity of hydrogels, various strategies have been explored. For ionic conductivity, ionic salts and polymers are incorporated into the matrix to facilitate effective ion transport. Polyelectrolyte hydrogels, due to their intrinsic charge, further enhance ion mobility. Achieving high ionic conductivity requires optimizing factors such as pore size and cross-linking density. For electronic conductivity, conductive fillers such as graphene, CNTs, and conductive polymers (e.g., PEDOT:PSS, polypyrrole (PPy)) are incorporated into the hydrogel matrix [73]. Conductive polymers play a crucial role in the manufacturing of hydrogels, particularly in enhancing their electrical conductivity, which is essential for bioelectronics and HMI applications. These polymers, such as PPy, polyaniline (PANI), and PEDOT, are incorporated into hydrogels to enable the transmission of electrical signals, allowing hydrogels to function in applications such as wearable sensors and neural interfaces. By providing electrical conductivity, conductive polymers facilitate the interaction between biological tissues and electronic devices, enabling real-time signal transmission. Additionally, they contribute to the biocompatibility and stability of hydrogels, ensuring they can interact with biological tissues for extended periods without adverse effects. Conductive polymers also improve the mechanical properties of hydrogels, such as elasticity and flexibility, which are vital for applications in soft robotics, haptic feedback systems, and flexible wearable electronics. Moreover, these polymers enable hydrogels to exhibit electroactive behavior, responding to electrical stimuli by changing their shape or properties, which is particularly advantageous in systems such as drug delivery devices and artificial muscles. Through these various roles, conductive polymers significantly enhance the functionality and versatility of hydrogels in advanced biomedical and electronic applications. To optimize conductivity while maintaining mechanical integrity, these fillers must be evenly distributed throughout the hydrogel matrix [73,74,75]. For instance, a temperature-triggered adhesive ionic conductive hydrogel composed of polyacrylamide, gelatin, and sodium alginate improves adhesion and conductivity at body temperature due to gelatin’s structural changes, with LiCl further enhancing ionic transport [76]. Similarly, incorporating polymers such as PEDOT:PSS and conductive fillers such as polyaniline (PANI) establishes a conductive network, optimizing electron transport. Several fabrication techniques have been employed to develop conductive polymer hydrogels with enhanced electrical and mechanical properties. These methods include in situ polymerization, electropolymerization, and hybrid material integration, each offering distinct advantages in tailoring conductivity, flexibility, and stability. As illustrated in Figure 3, these strategies enable the precise engineering of conductive hydrogels, making them suitable for applications ranging from bioelectronic interfaces to flexible sensors. Another innovative approach involves the use of liquid metal (LM)-doped hydrogels, which exhibit high electrical conductivity and toughness through self-sintering LM microdroplets [77]. These strategies align with the previous findings, reinforcing the role of ionic salts and polyelectrolytes in ionic conductivity and conductive fillers in electronic conductivity [78].

Several case studies highlight the successful application of conductive hydrogels in biomedical and electronic systems. For example, one study developed a conductive hydrogel-based pressure sensor for real-time monitoring of intraocular pressure (IOP) [79]. Another study by Lee et al. (2022) demonstrated the use of a conductive hydrogel for cardiac tissue repair [80]. The hydrogel composed of gelatin methacrylate (GelMA) and reduced graphene oxide (rGO) promoted cardiomyocyte alignment and electrical coupling, enhancing the functionality of engineered cardiac tissues [80]. Additionally, a recent study by Zhao et al. (2024) investigated a conductive organohydrogel electronic skin capable of detecting subtle pressure changes and temperature variations [81]. The electronic skin exhibited multimodal sensing properties with four modes: humidity, strain, bioelectric signals, and temperature.

Despite these advancements, challenges remain in optimizing the stability, biocompatibility, and conductivity of hydrogels without compromising their softness and stretchability. Long-term stability in physiological environments remains a key concern as conductive hydrogels may suffer from degradation, swelling, or loss of conductivity over time. Achieving a balance between mechanical robustness and electrical performance is essential to ensure the reliability of these materials in practical applications. Furthermore, biocompatibility must be carefully considered to minimize immune responses and ensure safe integration with human tissues. Future research should focus on the development of next-generation conductive hydrogels with enhanced durability, multifunctionality, and adaptability to dynamic biological environments. For example, integrating self-healing mechanisms and stimuli-responsive properties could further improve the performance of conductive hydrogels in HMI systems. Additionally, the use of machine learning to optimize material formulations and fabrication processes could address challenges related to reproducibility and scalability [82]. By addressing these challenges, conductive hydrogels have the potential to revolutionize HMI systems, paving the way for more advanced, seamless, and long-lasting interfaces between humans and machines.

Conclusively, conductive hydrogels represent a transformative material for HMI applications, offering a unique combination of electrical conductivity, mechanical flexibility, and biocompatibility. Their applications in neural interfaces, biosensors, and wearable electronics demonstrate their potential to overcome the limitations of traditional rigid materials. Advanced fabrication techniques, such as the incorporation of ionic salts, conductive fillers, and hybrid materials, have further enhanced their performance. However, challenges related to long-term stability, mechanical–electrical balance, and biocompatibility must be addressed to fully realize their potential. By leveraging emerging strategies and technologies, conductive hydrogels are poised to play a pivotal role in developing next-generation HMI systems, enabling more seamless and effective integration between humans and machines.

### 3.2. Hybrid and Composite Hydrogels

Hybrid and composite hydrogels are advanced materials formed by combining hydrogel matrices with nanomaterials, synthetic polymers, or bioactive agents to enhance their mechanical, electrical, and functional properties [82]. These hydrogels offer significant improvements in terms of versatility and performance, making them suitable for a wide range of applications in fields such as biomedical devices, soft robotics, and sensor technologies. By incorporating nanoparticles, polymers, and biologically active components, hybrid and composite hydrogels can exhibit enhanced mechanical strength, biocompatibility, and responsiveness to external stimuli, allowing for tailored designs that meet specific requirements for various applications [86].

There are several types of hybrid hydrogels. Nanocomposite hydrogels are created by integrating nanomaterials such as CNTs, graphene, MXenes, or ceramic nanoparticles into the hydrogel matrix [87]. These materials enhance the mechanical strength, electrical conductivity, and responsiveness of the hydrogels, making them ideal for bioelectronics, sensors, and actuation devices [88]. Polymer hybrid hydrogels, on the other hand, involve blending synthetic and natural polymers such as polyethylene glycol (PEG)–alginate or chitosan–PVA to optimize biofunctionality [89]. These combinations allow for improved biodegradability, cell adhesion, and mechanical stability, making them valuable in drug delivery, wound healing, and tissue engineering applications. Bioactive hydrogels are functionalized with bioactive molecules, such as peptides, proteins, or drugs, to improve cell interactions and biointegration, enhancing their use in regenerative medicine and controlled drug delivery systems [90].

Hybrid and composite hydrogels have led to key innovations and applications in several fields. They are being used in soft robotics and artificial muscles due to their ability to mimic the flexibility and stretchability of biological tissues, with the added advantage of responsiveness to external stimuli such as electrical fields and temperature changes [91]. Additionally, biodegradable and transient hydrogels are being developed for implantable bioelectronics, where they provide temporary support and degrade safely within the body after serving their purpose. These hydrogels also show promise in the development of smart wound dressings that offer antimicrobial properties, promote healing, and release bioactive agents in response to environmental changes such as infection or pH levels [92].

Despite their promising potential, hybrid and composite hydrogels face challenges in scalability, mechanical stability, and uniform nanoparticle dispersion. Scaling up the production of these hydrogels to industrial levels can be complex as ensuring uniform incorporation of nanoparticles or bioactive agents in the hydrogel matrix is crucial for maintaining their desired properties [93]. Furthermore, achieving the required mechanical strength and stability for prolonged use in biomedical or wearable applications remains a significant challenge. Addressing the issue of uniform nanoparticle dispersion is also essential, as poor distribution can lead to aggregation, diminishing the hydrogel’s overall performance. Nonetheless, ongoing research and technological advancements continue to address these challenges, paving the way for the widespread use of hybrid and composite hydrogels in diverse applications.

### 3.3. Fabrication Techniques

The development of complex hydrogel structures for sophisticated HMI applications relies on advanced fabrication techniques that enable precise control over their structural, mechanical, and functional properties. Three-dimensional printing methods, such as stereolithography (SLA) and inkjet printing, have emerged as powerful tools for fabricating hydrogel structures with customized geometries and functionalities. These techniques allow for the creation of patient-specific designs, making them ideal for applications in tissue engineering, biosensors, and soft robotics. Additionally, microfabrication techniques, including photolithography and microcontact printing, enable the creation of nanoscale features within hydrogels, enhancing interfacial properties and cellular interactions [93]. These methods are particularly valuable for neural interfaces and microfluidic devices, where precise control over surface topography and chemical patterning is critical.

The fabrication of conductive, hybrid, and composite hydrogels further expands the possibilities for HMI applications by integrating advanced materials and techniques. For instance, the freeze–thaw method has been utilized to synthesize multimodal transparent conductive hydrogel electrodes, such as PVA@HACC@HA, which exhibit enhanced mechanical stability and transparency [94]. This method ensures uniform distribution of conductive components while maintaining the hydrogel’s structural integrity. Similarly, dopamine-limited area polymerization has been applied in the synthesis of dPEDOT–CA–PDA–PAM hydrogels for BMIs, offering controlled polymerization for improved conductivity and adhesion [95]. These hydrogels are particularly promising for neural recording and stimulation due to their biocompatibility and electrical performance.

Moreover, electrochemical deposition and chemical cross-linking have been employed to develop PEDOT: poly (SS-4VP) interpenetrating network hydrogels, which provide stable neural interfaces with long-term conductivity [96]. These techniques enable the integration of conductive polymers into hydrogel matrices, enhancing their electrical properties without compromising their mechanical flexibility. These approaches complement the existing fabrication techniques, such as 3D printing and microfabrication, by providing additional pathways to tailor hydrogel properties for specific applications. For example, SLA and inkjet printing allow for the creation of customizable 3D hydrogel structures, while photolithography and microcontact printing enable the fabrication of nanoscale features that enhance interfacial interactions [97].

The integration of these advanced fabrication techniques is essential for designing hydrogels with enhanced mechanical, electrical, and biological properties, expanding their applicability in biomedical and electronic systems [98]. For instance, combining 3D printing with self-assembly or electrochemical deposition could enable the creation of hydrogels with hierarchical structures and multifunctional capabilities. Similarly, the use of machine learning to optimize fabrication parameters can improve reproducibility and scalability, addressing the key challenges in mass production [99].

**Figure 4:**
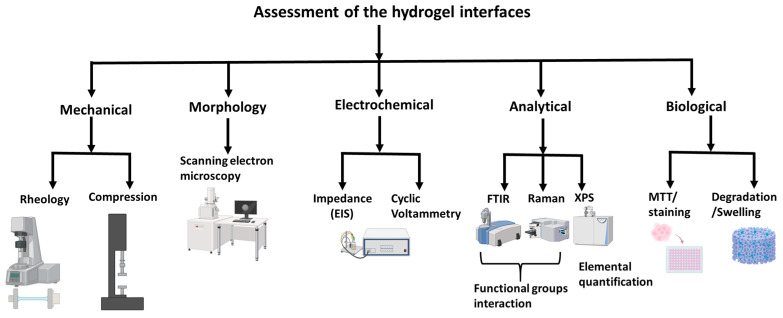
Evaluation and assessment of the hydrogel biointerfaces, including mechanical testing, morphological analysis, electrochemical assessments, chemical composition, biocompatibility, and stability studies.

As these technologies continue to evolve, they will pave the way for innovative solutions in health care, robotics, and beyond, enabling the development of adaptive, responsive, and biocompatible interfaces. Conclusively, the fabrication of hydrogels for HMI applications has been revolutionized by advanced techniques such as 3D printing, microfabrication, and specialized methods such as freeze–thaw, dopamine-limited polymerization, and electrochemical deposition. These approaches enable the creation of hydrogels with tailored properties, making them suitable for a wide range of applications, from neural interfaces and biosensors to soft robotics and tissue engineering. By integrating these techniques and addressing challenges such as reproducibility and scalability, researchers can further expand the potential of hydrogels in bridging the gap between machines and biological systems. A summary of the key evaluation and assessment techniques for these fabricated hydrogels is provided in Figure 4.

## 4. Polymer Classes Modulating Electrical Conductivity in Hydrogels

The incorporation of conductive materials into hydrogels is fundamental to developing biointerfaces for biomedical, wearable, and electronic applications. With their high water content, flexibility, and biocompatibility, hydrogels are well-suited for environments requiring soft and stretchable materials [100]. However, to enhance their electrical properties for applications such as neural interfaces, biosensors, and flexible electronics, conductive polymers are often integrated into the hydrogel networks. The selection of polymers is critical in determining the mechanical, electrical, and chemical characteristics of the resulting hydrogel. Various polymer classes, including natural, synthetic, conductive, and nonconductive, can be employed to optimize the hydrogel’s conductive behavior. This section explores the role of different polymer types in fabricating conductive hydrogels, highlighting their impact on mechanical stability, electrical performance, and overall functionality.

**i.**  **Conductive polymers**

Conductive polymers, including PPy, PANI, and PEDOT, have been widely explored for their ability to introduce electrical conductivity into hydrogel systems. These polymers possess conjugated π-electron systems that enable efficient electron transport along their molecular chains. When integrated into hydrogels, they provide a conductive network while preserving the material’s intrinsic hydrophilicity [101]. The incorporation of these polymers significantly enhances hydrogel performance in applications requiring efficient signal transmission, such as neural interfaces, biosensors, and electroactive devices.

Polypyrrole (PPy): PPy is among the most extensively studied conductive polymers due to its ease of synthesis and environmental stability. PPy-based hydrogels are typically fabricated through in situ polymerization, where pyrrole monomers polymerize within the hydrogel matrix [102]. Conductivity can be further improved through doping with counterions such as tosylate or sulfate, which facilitate charge transport [103]. PPy-based hydrogels exhibit favorable mechanical resilience and biocompatibility, making them well-suited for applications in neural interfaces and soft robotics.

Polyaniline (PANI): PANI stands out due to its tunable conductivity, which depends on its oxidation state, making it a highly versatile material for sensors and actuators. When incorporated into hydrogels, PANI enhances electrochemical performance while maintaining the flexibility needed for wearable sensing platforms [104]. PANI-based hydrogels are particularly advantageous in applications where materials must withstand mechanical deformations while preserving conductivity, ensuring reliable performance in dynamic environments.

Poly(3,4-ethylenedioxythiophene) (PEDOT): PEDOT, commonly formulated with PSS as PEDOT:PSS, is a widely utilized conductive polymer in flexible and stretchable electronics. PEDOT-based hydrogels offer superior electrical conductivity and long-term stability, making them particularly valuable in tissue engineering and biosensing applications where both electrical and biological compatibility are essential. The inclusion of PEDOT enhances hydrogel conductivity without significantly compromising mechanical flexibility, enabling its use in advanced bioelectronic systems [105].

Polyacetylene (PAc): Polyacetylene is one of the earliest discovered conductive polymers due to its conjugated π-electron system [106]. While its conductivity is relatively low compared to other conductive polymers such as PEDOT or PANI, it can still be incorporated into hydrogels to enhance their electrical properties. Polyacetylene-based hydrogels are typically doped with dopants (such as iodine or halogens) to improve their conductivity [107].

Polythiophene (PTh): Polythiophene is another highly conductive conjugated polymer. It is more stable than polyacetylene and has excellent electrical properties, making it a promising candidate for use in conductive hydrogels. Polythiophene can be incorporated into hydrogels to improve their conductivity, flexibility, and biocompatibility, which are particularly important for applications in flexible electronics, biosensors, and neural interfaces [108].

**ii.**  **Natural polymers**

Natural polymers, such as polysaccharides, proteins, and nucleic acids, play a critical role in the synthesis of conductive hydrogels due to their inherent biocompatibility, biodegradability, and functional versatility. These polymers offer a sustainable alternative to synthetic counterparts and can be tailored to improve both the mechanical and electrical properties of hydrogels [106]. Their ability to be chemically modified or combined with conductive materials makes them highly suitable for applications in tissue engineering, biosensing, and bioelectronic interfaces.

Alginate: Alginate, a naturally derived polysaccharide extracted from brown seaweed, is extensively used in hydrogel formulations due to its biocompatibility and ease of gelation through ionic cross-linking with divalent cations (e.g., Ca^2+^). While alginate itself is electrically insulating, its conductivity can be significantly enhanced by incorporating conductive fillers such as PEDOT, graphene oxide (GO), or CNTs [109]. Alginate-based conductive hydrogels have been employed in neural interfaces, biosensors, and soft bioelectronics due to their ability to provide a hydrated, ionically conductive matrix suitable for interfacing with biological tissues.

Chitosan: Chitosan, a cationic polysaccharide derived from chitin, exhibits biodegradability, antimicrobial properties, and the ability to form stable hydrogels. Its amino-functionalized backbone allows for chemical modification and interaction with other conductive polymers [110]. The incorporation of these materials enhances chitosan hydrogels’ electrical conductivity, making them suitable for wound healing, electroactive scaffolds, and nerve regeneration applications. Furthermore, chitosan’s ability to chelate metal ions facilitates additional conductivity enhancement through metal nanoparticle incorporation.

Gelatin: Gelatin, a protein-based biopolymer derived from collagen, is widely used in hydrogel fabrication due to its capacity to mimic the extracellular matrix (ECM), supporting cellular adhesion, proliferation, and differentiation. While inherently nonconductive, gelatin-based hydrogels can be rendered conductive by incorporating PANI into its backbone through genipin-induced cross-linking [111] and PEDOT:PSS [112]. These hybrid hydrogels have shown promise in bioelectronics, neural interfaces, and cardiac tissue engineering, where electrical stimulation is crucial for cellular communication and function.

By harnessing the distinctive characteristics of natural polymers and incorporating conductive elements, hydrogels can be designed to simultaneously offer structural integrity and electrical conductivity, broadening their applicability in advanced biomedical devices and bioelectronic systems.

**iii.**  **Synthetic polymers**

Synthetic polymers are widely utilized in the development of conductive hydrogels due to their customizable properties and ease of functionalization. These materials offer the ability to fine-tune mechanical, electrical, and thermal characteristics, making them highly suitable for applications in flexible electronics, wearable sensors, and biomedical devices.

Polyvinyl alcohol (PVA): PVA is a synthetic polymer commonly used in hydrogel fabrication due to its excellent water absorption capacity and biocompatibility. When integrated with conductive materials such as PEDOT:PSS, PVA-based hydrogels exhibit improved electrical conductivity [112]. Additionally, PVA enhances the mechanical robustness and thermal stability of hydrogels, making them suitable for long-term biomedical and electronic applications.

Polyethylene glycol (PEG): PEG is another widely employed synthetic polymer in hydrogel design. While PEG itself lacks intrinsic conductivity, it can be chemically modified with conductive moieties or combined with conductive fillers such as GO or CNTs to enhance its electrical properties [113]. Due to its high water content and tunable mechanical properties, PEG-based hydrogels are frequently used in drug delivery systems, biosensors, and soft electronic devices.

Polyacrylamide (PAAm): PAAm hydrogels are known for their high mechanical resilience, making them ideal for applications requiring structural integrity under stress. Although inherently nonconductive, PAAm hydrogels can be engineered for conductivity by incorporating conductive nanoparticles or polymers. These modifications make PAAm hydrogels valuable in bioelectronics, stretchable sensors, and electronic skin, where both durability and electrical performance are essential [114].

**iv.**  **Hybrid polymers for conductive hydrogels**

Hybrid polymers, which combine synthetic and natural polymeric components, offer a versatile approach to hydrogel design by integrating the advantages of both material classes. These composites often exhibit superior mechanical strength, enhanced conductivity, and improved biocompatibility, expanding their potential applications in wearable bioelectronics and implantable devices [115].

Graphene-based hybrid hydrogels: Graphene-based materials, including GO and rGO, serve as effective conductive fillers in hydrogel matrices. When combined with polymers such as PVA or alginate, these hybrids significantly improve the electrical conductivity of hydrogels while preserving mechanical flexibility [116]. This makes them particularly beneficial for applications in biosensing, bioelectronics, and wearable technology.

**Figure 5:**
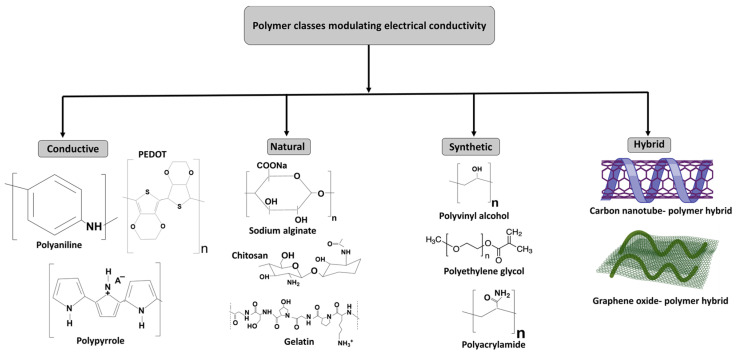
Various types of polymers modulating electrical conductivity, categorized into conductive, natural, synthetic, and hybrid polymers, that play a crucial role in optimizing the conductive properties of hydrogel biointerfaces for HMI applications.

Carbon nanotube (CNTs)-based hybrid hydrogels: CNTs are widely recognized for their exceptional electrical conductivity and mechanical strength. Incorporating CNTs into hydrogel networks enhances their electroconductive properties, making them well-suited for advanced applications such as neural interfaces, soft robotics, and artificial muscles [65]. The combination of CNTs with biocompatible polymers allows for the development of hydrogels that maintain flexibility while ensuring efficient electrical signal transmission. Different types of polymers that are used in fabricating conductive hydrogels are shown in Figure 5.

## 5. Spectrum of the Mechanisms of Hydrogel–Machine Interfacing

The interaction between the human body and machines is built on a range of communication mechanisms, each designed for specific applications and the distinct properties of biological tissues. Hydrogels have become a key component in these interfaces, offering the flexibility to combine multiple functional modes to meet the complex demands of these interactions. Based on their interaction mechanisms, hydrogel interfaces can be categorized into six primary functional modes: mechanical, acoustic, electrical, optical, chemical, and biological. The ability to integrate these diverse functions is critical for the effective synchronization of devices that operate across multiple domains simultaneously. For example, epidermal electrodes require hydrogel interfaces that provide electrical conductivity while also exhibiting mechanical properties such as flexibility, stretchability, and adhesion to ensure seamless integration with the skin. Similarly, hydrogels used in sensing, actuation, or therapeutic applications may need to simultaneously exhibit optical, chemical, or biological properties alongside their mechanical or electrical characteristics. Hydrogel interfaces must integrate multiple properties to enhance functionality in human–machine interactions. They require mechanical flexibility, strong adhesion, and durability for seamless biological integration. Acoustic and electrical properties ensure efficient energy transfer, signal stability, and ion transport. Optical responsiveness enables controlled light interaction, while chemical selectivity supports molecular recognition and catalysis. Biocompatibility is essential for long-term stability and cellular interaction. By combining these attributes, hydrogels become ideal for wearable and implantable bioelectronic applications [117].

In terms of biological interactions, the biological mode of hydrogel interfaces is critical for ensuring the efficacy, reliability, and biosafety of devices, particularly those that are more invasive or interact with the body for extended periods. Biological responses at these interfaces greatly influence the performance of hydrogels in vivo and can be divided into two categories based on application needs: promoting beneficial biological activities (such as cell adhesion, proliferation, and differentiation) and preventing undesirable biological responses (such as foreign body reactions). For implantable applications, such as drug delivery or tissue repair devices, the infiltration of cells from surrounding tissues and the gradual remodeling of the hydrogel by native cells are essential for long-term success. For example, tissue adhesives and sealants require cellular infiltration, followed by hydrogel degradation and tissue remodeling, to avoid the need for surgical removal and minimize the risk of chronic inflammation. The key properties such as high cell adhesiveness and biodegradability are essential for promoting tissue integration and remodeling in such applications [118,119]. On the other hand, regulating unwanted biological interactions—such as biofouling or foreign body responses—is crucial for enhancing the longevity and effectiveness of hydrogel interfaces. These undesirable responses often lead to fibrosis, which can impair device function by reducing electrical conductivity or chemical diffusivity, negatively impacting the efficacy of electrical sensing, stimulation, and chemical delivery. Minimizing foreign body responses is especially important for ensuring the long-term effectiveness of hydrogel interfaces used in electrical, chemical, and optical applications [120,121].

The mechanical properties of hydrogel interfaces play an essential role in maintaining their structural integrity, robustness, and stable adhesion to biological tissues and organs, especially in long-term applications. These properties are crucial for wearable health-monitoring devices, which remain in close contact with the skin for extended periods, as well as implantable devices that may stay in contact with internal tissues for months or years [122]. Because the stiffness of tissues and organs varies, mismatched stiffness between hydrogels and their target tissues can result in impaired functionality, poor contact, and long-term tissue responses such as inflammation, foreign body reactions, and scarring. To mitigate these risks, hydrogel interfaces should have a mechanical stiffness, or Young’s modulus, comparable to the target tissue. Additionally, hydrogel interfaces must withstand varying mechanical forces and deformations over time, especially as the body is constantly subjected to mechanical stress. For applications such as epidermal electrodes, tissue adhesives, and sealants, robust adhesion is crucial to prevent device failure or detachment, which could result in a loss of function or clinical complications. Furthermore, minimizing friction at the tissue–hydrogel interface is key to avoiding damage or wear, ensuring durability, and preventing issues such as erosion or tissue damage caused by prolonged friction [123,124].

Ultrasound-based diagnostics have become integral in clinical settings due to their noninvasive nature, providing valuable insights into diseases and health conditions. Ultrasound technology is also widely used in therapeutic applications, including thermal treatments and drug delivery. Hydrogel interfaces play a dual role in ultrasound-based applications: in acoustic imaging, where the hydrogel aids in delivering sound waves to the target and receiving reflections (e.g., in ultrasonography), and in acoustic stimulation, where sound waves are delivered to the target (e.g., in ultrasonic lithotripsy). Since the impedance of ultrasound transducers differs significantly from the acoustic impedance of human tissue, hydrogel interfaces that ensure tissue-matching acoustic impedance and conformal contact with the skin are highly advantageous, improving the efficiency of sound wave transmission and the quality of diagnostic imaging [125].

The optical mode of hydrogel interfaces is focused on the delivery of light to the human body. This is particularly important in ophthalmic applications, where hydrogel interfaces must allow the unobstructed transmission of light to the eye. Similarly, hydrogel interfaces are being used for light delivery in a variety of therapeutic and diagnostic applications, such as photonic treatments and therapies. For effective light delivery, hydrogel interfaces must have high transmittance and a tunable refractive index to control the transmission and internal reflection of light, enabling precise light delivery to targeted tissues [126,127].

Chemical interactions are another critical aspect of the hydrogel interface, given the complex chemical processes that occur within the body. The high water content of both biological tissues and hydrogel interfaces facilitates the exchange of waterborne chemicals, making the chemical mode of hydrogel interfaces particularly important. Chemical interactions with the human body can fall into two categories: chemical delivery from the hydrogel to the body and chemical sensing from the body to the hydrogel. Chemical delivery is often employed for administering pharmacological substances such as drugs and biologics. Since the effectiveness and toxicity of drugs are highly dependent on their dosage and release profile, controlled release from hydrogel interfaces is essential. The rate of chemical delivery can occur via diffusion or degradation of the hydrogel, with the diffusion rate depending on the diffusivity of the chemical within the hydrogel, while degradation-based delivery is influenced by the hydrogel’s biodegradability. For effective drug delivery, hydrogel interfaces must possess tunable properties such as mesh size, chemical interactions (e.g., binding and release), and biodegradability [32,128]. For chemical sensing, the hydrogel interface must selectively interact with target chemicals, with the binding affinity between the hydrogel and the chemical being crucial for efficient sensing. A higher binding affinity enhances the selectivity of the hydrogel for the target chemical, making it critical for effective chemical detection [129,130].

## 6. Hydrogel Interfaces for Seamless Human–Machine Integration

Before the advancement of hydrogel-based interfaces, the traditional materials used in HMI included rigid metallic electrodes (e.g., platinum, titanium, gold), silicon-based electronics, polymer-derived implants (e.g., PEG, polydimethylsiloxane, polyimide, and carbon-based materials (e.g., CNTs and graphene) [131,132,133]. While such traditional materials have played a crucial role in wearable sensors, bioelectronic devices, and medical implants, they often suffer from mechanical mismatches with soft human tissues due to limited biocompatibility and challenges related to long-term integration with biological systems [134,135]. On the other hand, such types of rigid devices may lead to inflammation, immune responses, and discomfort when interfaced with a soft, dynamic biological environment, thereby limiting their suitability for continuous or long-term biomedical applications [136,137]. Hydrogel interfaces have become a superior alternative to conventional biomaterials by addressing their limitations through providing improved biocompatibility, flexibility, and seamless integration for human–machine interactions, resulting in widespread adoption in diverse academic and commercial applications involving devices that interact with the human body [138,139]. Hydrogels, consisting of cross-linked hydrophilic polymeric networks that exhibit high water content, mechanical flexibility, physical, chemical, and mechanical biocompatibility, and responsiveness to external stimuli, enabling seamless integration with biological tissues, reduced mechanical stress and enhanced adhesion and adaptive contact for applications in biosensing, soft robotics, implantable medical devices, and tissue engineering [140,141]. The applications of hydrogel interfaces in human–machine interaction are categorized by their level of invasiveness, as shown in Table 1.

**Table 1:** Applications of hydrogels for human–machine interfacing.

Category	Application	Hydrogel Used	Level of Invasiveness	References
Epidermal and wearable	Ultrasound probe	Couplant for ultrasonic imaging		[138,142,143]
	Epidermal electrode	Skin contact applications		
	Wound dressing	Wound healing and moisture retention	Noninvasive communication	
	Sweat sensor	Monitoring sweat biomarkers		
	Contact lens	Soft contact lenses for comfort and biocompatibility		
Implantable	Implantable electrode	Deep-tissue or neural interfaces		[144]
	Tissue adhesive	Bonding tissues in surgical applications		[145]
	Drug delivery carrier	Controlled drug release within the body	Fully invasive (long-term contact with internal organs and tissues)	[146]
	Anti-FBR coating	Reduced foreign body response (FBR) to implants		[147]
	Optical waveguide	Light-based medical sensors		[148]
Ingestible	Ingestible device	Gastrointestinal monitoring and drug delivery		[149]
	Catheter	Reduce friction and enhanced biocompatibility		[150]
	Guidewire	Minimally invasive procedures	Minimally invasive in body cavities (thoracic and abdominal cavities) and tubular organs	[138]
	Stent	Blood vessel support and drug delivery		[138,151]

### 6.1. Epidermal and Wearable Applications

In the modern era, hydrogel-based materials are revolutionizing HMI by providing exceptional flexibility, biocompatibility, and functionality. These materials bridge the gap between biological tissues and electronic systems, enabling seamless integration for real-time health monitoring, diagnostics, and therapeutic interventions in wearable and epidermal applications. Their unique properties such as moisture retention, electrical conductivity, and the ability to conform to the body’s natural contours render hydrogels ideal for next-generation biomedical technologies. Among their most promising applications are epidermal electrodes, wound dressings, sweat sensors, contact lenses, and ultrasound probes, each playing a vital role in advancing personalized health care and interactive wearables.

The integration of hydrogels into ultrasound probe interfaces as coupling agents significantly enhances imaging quality and patient comfort. Unlike traditional ultrasound gels, which require frequent reapplication and may cause discomfort or inflammation, hydrogel-based coupling layers offer long-lasting adhesion and superior acoustic transmission, ensuring more stable and effective ultrasonic imaging [152,153]. For example, a modern wearable ultrasound patch has recently been developed, enabling continuous monitoring of internal organs, cardiovascular health, and fetal development without the need for repeated clinical visits [154,155,156]. Additionally, an advanced study demonstrated that hydrogel-coated ultrasound probes significantly reduce imaging artifacts and enhance acoustic impedance matching, thereby improving diagnostic accuracy. These breakthroughs in wearable and epidermal applications pave the way for real-time, noninvasive diagnostic tools that were previously unavailable [157,158]. Another key application of hydrogels in HMI is epidermal electrodes, which have revolutionized the capture of bioelectrical signals. Traditional metallic electrodes are dry and mechanically stiff, often leading to signal degradation, skin irritation, and discomfort during prolonged use. To overcome these challenges, hydrogel interfaces were introduced as coatings on metal electrodes, becoming the standard for various clinical diagnostics. These coatings provide exceptional skin conformity, enhanced conductivity, and stable long-term wear, making them ideal for EMG, ECG, and EEG applications [138,159]. Hydrogel-based ECG electrodes have been shown to significantly reduce motion artifacts compared to conventional adhesive electrodes, making them ideal for continuous monitoring in epidermal and wearable health devices [160,161]. These stretchable and flexible electrodes have unlocked new possibilities in wearable bioelectronics and enable users to monitor their health effortlessly while maintaining mobility [34].

Hydrogels also have broad applications in wound care management, offering superior healing environments compared to traditional dressing practices. They can maintain optimal moisture levels, reduce inflammation, and deliver therapeutic agents directly to the wound site, thereby improving the healing process while minimizing infections [162,163]. The development of smart hydrogel wound dressings with embedded biosensors has further advanced wound care by enabling real-time monitoring of pH levels, temperature fluctuations, and bacterial activity. This innovation not only reduces the need for frequent dressing changes, but also facilitates personalized, data-driven treatment strategies for patients with chronic wounds and burns [164,165]. For instance, a hydrogel dressing incorporated with antimicrobial peptides exhibited improved bacterial inhibition and accelerated healing times when compared to traditional gauze dressings [166,167].

Hydrogel-based sweat sensors represent an exciting development in wearable healthcare devices, enabling noninvasive analysis of the key biomarkers such as lactate, electrolytes, and glucose. Sweat, often underutilized as a biofluid, offers valuable insights into an individual’s metabolic state, hydration levels, and overall health [168,169]. By integrating hydrogel sensors into wearable patches and smart wristbands, researchers have developed innovative diagnostic tools that provide real-time feedback for athletes, diabetics, and individuals at risk of dehydration [170]. For instance, a hydrogel-based sweat sensor capable of real-time glucose monitoring in diabetic patients has been reported, reducing the need for invasive blood sampling. Such devices mark a significant advancement in continuous, noninvasive health monitoring, reducing reliance on traditional blood-based testing [168,171]. Perhaps the most futuristic application of hydrogels is smart contact lenses, which enable ocular health monitoring and ocular-targeted drug delivery [143]. Unlike conventional lenses, hydrogel-based smart lenses can also detect tear-fluid biomarkers related to conditions such as diabetes, glaucoma, and dry eye syndrome, allowing for early disease detection and proactive treatment [172,173]. Additionally, these lenses can be engineered to monitor IOP continuously, releasing disease-tailored on-demand medication [173]. Recent studies have demonstrated that hydrogel contact lenses embedded with nanocarriers were able to release anti-glaucoma drugs in a controlled manner, significantly improving patient compliance compared to traditional eye drops [174,175]. Incorporating wireless data transmission into these lenses boosts their capability to provide real-time updates to healthcare professionals, facilitating personalized treatment adjustments and enabling remote monitoring. The integration of hydrogel-based materials into wearable and epidermal HMIs has opened up new possibilities for seamless interaction, transforming health care with real-time, personalized, and noninvasive monitoring solutions for a wide range of medical conditions. Table 2 evaluates clinically approved hydrogel-based interfaces, emphasizing their applications in medical diagnostics and patient care.

**Table 2:** Commercial products utilizing hydrogel interfaces in neuroprosthetics, brain–machine interfaces, and neural signal transduction.

Product	Application	Hydrogel Use	Refs.
Neuralink	Implantable brain–computer interface (BCI)	Biocompatible hydrogel coating for electrode stability and decreased immune response	[176]
Medtronic Deep Brain Stimulation (DBS) Systems	Neuromodulation, Parkinson’s disease, and epilepsy treatment	Soft electrode coatings with potential hydrogel applications for improved conductivity and tissue integration	[122]
Cortec Neuroprosthetic Electrodes	High-resolution neural recording and stimulation	Hydrogel-modified electrodes being researched for stable neural signal transmission	[177]
BrainCo-BMI Headset	Noninvasive brain–computer interface	Hydrogel-based EEG electrodes for improved signal detection and comfort	[94]
Synchron Stentrode	Implantable neural interface for paralyzed patients	Exploring hydrogel coatings for electrode longevity and reduced inflammation	[122]
Bio-Signal Technologies EEG and EMG Electrodes	Neural signal acquisition for rehabilitation	Soft hydrogel interfaces for high- fidelity signal recording	[65,122]

### 6.2. Implantable Applications

Conventional implantable devices face significant challenges in HMI applications, mainly due to biocompatibility issues with traditional metallic and polymer-based electrodes. These materials often trigger immune responses, leading to inflammation, fibrotic encapsulation, and performance degradation [178,179]. Foreign body responses reduce signal transmission efficiency, accelerate device failure, and exacerbate the mechanical mismatch between rigid implants and soft tissues, compromising signal fidelity and stability [180]. Additionally, limited device lifespan due to material degradation and immune-mediated deterioration requires frequent replacement and maintenance [181]. Moreover, poor adhesion of conventional implants to biological tissues leads to unstable interfaces, impairing signal acquisition and reducing device efficiency. To address these issues, hydrogel-based interfaces offer a promising alternative, providing flexibility, biocompatibility, and seamless integration with biological tissues for long-term implantation [182].

As implantable electrodes, hydrogels offer highly conductive, bio-friendly environments, enhancing signal transmission and minimizing immune responses [183]. The hydrated, soft nature of hydrogels mimics natural tissues, reducing mechanical mismatch and ensuring stability in neural and electrophysiological recordings [184]. Hydrogels also function as effective tissue adhesives in implanted devices, offering strong, reversible bonding with biological surfaces, particularly in surgical procedures, enhancing attachment and reducing displacement [185,186]. Additionally, hydrogels serve as drug delivery carriers, enabling sustained, localized release for targeted therapies and improving the efficacy of implantable medical devices [187,188]. Hydrogels also serve as anti-FBR coatings, incorporating immune-modulating agents to reduce inflammation and enhance the long-term performance of implantable HMI systems [189,190].

Finally, hydrogel-based optical waveguides offer transparent, flexible, and biocompatible interfaces for optogenetic stimulation and neural imaging, allowing precise light transmission with minimal tissue damage [183,191]. These advances make hydrogel-based interfaces a transformative solution in implantable HMI technologies, enhancing biocompatibility, multifunctionality, and mechanical adaptability for long-term biomedical applications [164,192].

### 6.3. Minimally Invasive Applications

Traditional invasive medical devices, such as ingestible devices, guidewires, catheters, and stents, have been used for diagnostics and medical treatments. However, these devices often suffer from mechanical rigidity, biocompatibility issues, and tissue irritation, leading to complications such as inflammation, discomfort, and infection. Devices made from metals and rigid polymers do not conform to soft biological tissues, causing a mechanical mismatch and increasing the risk of injury at the application site [138,193]. Additionally, these devices lack adaptability and require surgical retrieval due to uncontrolled degradation byproducts [194,195].

To overcome these limitations, hydrogels present a promising alternative due to their high water content, tunable mechanical properties, and superior biocompatibility [196]. Ingestible hydrogel-based devices provide an advanced drug delivery platform capable of swelling in response to gastric fluids for controlled release and safe degradation. These devices improve gastrointestinal retention and reduce the frequency of drug administration, though maintaining stability across varying pH levels remains challenging [169,197,198]. Hydrogel-coated catheters enhance patient compliance by minimizing friction and reducing infection risks. Their lubricious properties facilitate smoother insertion, while antimicrobial-infused hydrogels prevent biofilm formation. However, degradation over time may compromise their effectiveness, necessitating material optimization [197,199]. Similarly, hydrogel-coated guidewires improve maneuverability during vascular interventions, reducing endothelial trauma and enhancing navigation through complex anatomical structures [200,201]. Some hydrogels also exhibit stimuli-responsive properties that adapt to physiological conditions, but their long-term stability under shear stress remains a concern [202]. Hydrogel-integrated stents address complications such as restenosis by providing a softer, more biocompatible interface that can release therapeutic agents to prevent excessive tissue proliferation. Some hydrogel-based stents have shape-memory properties, allowing controlled expansion while reducing mechanical stress. However, structural integrity under prolonged mechanical loads must be improved before widespread clinical application [203,204]. Despite their advantages, hydrogel-based minimally invasive devices face challenges in stability, mechanical durability, and sterilization. Further research is necessary to optimize these materials for long-term applications and regulatory approval [205,206].

### 6.4. Neural and Bioelectronic Interfaces

Hydrogel interfaces in HMI have become a transformative technology that bridges the gap between biological tissues and artificial systems due to their hydrophilic and soft mechanical properties, making them ideal for integrating with natural tissues and electronics [179,207]. While traditional rigid bioelectronic materials face integration challenges, hydrogels offer tunable biocompatibility, improved ionic conductivity, and flexibility, enabling them to seamlessly adapt to dynamic biological structures, making them ideal for long-term bioelectronic applications [138,208]. In the domain of neural and bioelectronic interfaces, hydrogels have shown remarkable potential. Their soft, hydrated nature reduces mechanical mismatches between implanted devices and delicate neural tissues, minimizing inflammatory responses and enhancing the stability of bioelectronic implants. Moreover, the ionic properties of hydrogel interfaces facilitate efficient charge transport, essential for neural signal transmission, making them ideal for applications in BMIs, neuroprosthetics, and neural signal transduction [209,210].

In neuroprosthetics, hydrogel interfaces serve as intermediates between neural tissues and prosthetic devices, improving signal acquisition and biocompatibility while addressing the long-term stability challenges of traditional neural prostheses, which often suffer from tissue scarring and electrode degradation. Hydrogel coatings on neural electrodes improve implant longevity by reducing immune responses and maintaining stable electrical conductivity [211,212]. For instance, a study demonstrated that hydrogel coatings on neural electrodes significantly improved long-term stability in neural recordings by reducing fibrotic tissue formation [211].

**Figure 6:**
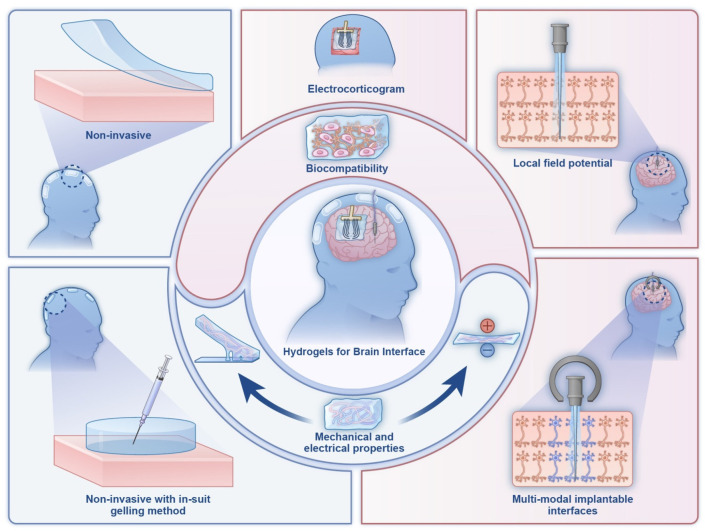
Application of hydrogels in brain signal monitoring. Hydrogels improve neural signal acquisition and transmission in both invasive and noninvasive BMI systems, facilitating the recording of electrocorticography and local field potential signals through invasive neural electrodes. Reproduced from Ref. [94] under the Creative Commons Attribution 4.0 International License.

BMIs have seen substantial advancements with the integration of hydrogel materials, facilitating bidirectional communication between external devices and neural circuits. This enables seamless control of prosthetic limbs, communication aids, and neurorehabilitation systems [213]. A recent study introduced a hydrogel-based artificial neuron capable of chemically mediated synaptic transmission, enhancing the naturalistic function of BMIs by improving signal transduction and synaptic plasticity [214]. Furthermore, hydrogel-based, noninvasive BMI electrodes improve EEG signal quality, demonstrating applications both in invasive and noninvasive BMI systems [215]. Figure 4 illustrates the application of hydrogels in brain signal monitoring, emphasizing their role in improving neural signal acquisition and transmission in both invasive and noninvasive BMI systems. Invasive neural electrodes facilitate the recording of electrocorticography (ECoG) and local field potential (LFP) signals, as shown in Figure 6.

Neural signal transduction is a key application of hydrogel interfaces as they enhance neural communication by improving charge transport at the electrode–tissue interface. Conventional silicon-based or metallic neural interfaces often suffer from impedance mismatches, leading to signal degradation. In contrast, hydrogel interfaces provide a conductive, hydrated medium that supports more stable and efficient neural signal transmission. Studies have explored the integration of hydrogels into electronic tissue technologies, demonstrating their seamless incorporation into neural network systems while maintaining high signal transduction [216]. Similarly, research has highlighted the potential of bioadhesive hydrogel-integrated BMIs for long-term neural recording and stimulation, demonstrating their ability to maintain signal integrity in complex neuroelectronic devices [217].

Given their diverse applications in neuroprosthetics, BMIs, and neural signal transduction, hydrogel-based interfaces hold great promise in transforming the field by providing biomimetic and biocompatible solutions for long-term neural interfacing. However, challenges remain, particularly concerning material stability and electrical conductivity. Further research is needed to focus on developing multifunctional hydrogel systems that incorporate conductive nanomaterials and bioactive molecules, enhancing their therapeutic potential in neural engineering applications. Moreover, the integration of hydrogels with bioelectronics and neurotechnology is set to drive groundbreaking innovations, laying the foundation for more effective and reliable HMI applications.

### 6.5. Soft Robotics and Actuators

Hydrogels have become a fundamental material in HMI, particularly in soft robotics and actuators, due to their high biocompatibility, tunable mechanical properties, ionic conductivity, and ability to retain high water content while maintaining structural integrity. These features allow for seamless integration into biological and bioinspired robotic systems [93,218]. Hydrogel-based materials also enable the development of advanced artificial muscles and robotic actuators that mimic natural biological functions, making them essential components in next-generation wearable robotics and bioelectronics [219]. Their soft, adaptable nature increases the functionality and comfort of wearable robotic devices, making them highly desirable for soft robotic actuators and artificial muscle applications [220,221].

Recent advancements in hydrogel ionotronics have enabled their use in soft robotic systems as sensors and actuators [222]. Studies have demonstrated that hydrogel-based ionotronic devices enhance HMI by enabling real-time responsiveness in wearable robotic applications, contributing to the development of next-generation robotic exoskeletons and prosthetic systems through improved mechanical adaptability and signal transduction efficiency [221,223,224]. The incorporation of ionically conductive hydrogels in soft robotic actuators further enhances sensitivity and real-time feedback, essential for developing adaptive robotic instruments.

Hydrogel interfaces are also key in the development of dielectric elastomer actuators (DEAs), which use highly stretchable and conductive hydrogel layers as electrodes. These actuators deform under an applied electric field, mimicking the contraction and relaxation of natural muscle fibers [225,226]. Unlike conventional rigid actuators, hydrogel-based DEAs provide smooth and controlled motion, making them suitable for robotic grippers, prosthetics, and bioinspired locomotion systems [227,228]. Hydrogel–DEA systems have been successfully implemented in soft robotic hands to facilitate precise and adaptive grasping motions [218].

Thermoresponsive hydrogels, which exhibit reversible swelling and a shrinking behavior in response to temperature changes, have also been developed for soft robotics. These hydrogels serve as stimuli-responsive actuators, allowing robotic systems to adjust their configuration based on environmental temperature fluctuations. For example, hydrogel-based grippers can autonomously open and close in response to ambient temperature changes, offering promising solutions for biomedical applications such as minimally invasive surgeries and drug delivery systems [229,230]. Self-healing hydrogels, inspired by biological tissues, are being explored to enhance the durability of soft robotic systems. These hydrogels autonomously repair minor structural damage, ensuring long-term stability in dynamic environments, such as wearable robotics and bioelectronic devices [231,232]. Self-healing hydrogels with integrated conductive networks have been successfully implemented in artificial muscle systems, enabling prolonged usability without performance degradation [231]. Additionally, nanocomposite hydrogels, incorporating nanomaterials such as graphene, metal nanoparticles, and CNTs, have improved the electrical and mechanical properties of hydrogel-based actuators, leading to superior electromechanical performance and efficient movement in robotic systems [233,234].

Despite their numerous advantages, certain challenges still hinder the widespread adoption of hydrogel-based interfaces in HMI applications. Long-term stability, mechanical durability, and response time must be addressed to enhance the practicality of hydrogel-based actuators [159]. Efforts in polymer engineering and material science are continuously being made to overcome these limitations, with ongoing research focusing on developing hydrogels with enhanced toughness, rapid response characteristics, and long-term durability.

### 6.6. Haptics and Sensory Systems

Hydrogel-based interfaces have emerged as transformative materials in human–machine interaction (HMI), particularly in advancing haptic technology and sensory feedback systems, thanks to their mechanical flexibility, biocompatibility, and ionic conductivity. These properties offer an ideal platform for developing artificial skin, electronic touch sensors, and soft actuators, making hydrogels essential for enhancing haptic applications in augmented reality (AR), virtual reality (VR), and biomedical prosthetics [159,235,236]. Hydrogels can mimic human skin properties, enabling seamless integration into haptic systems to improve tactile feedback mechanisms and allowing users to interact with digital environments in a more immersive way by transmitting multimodal sensory information, such as pressure, temperature, and texture variations [237,238].

A significant challenge in traditional haptic technology is replicating realistic touch sensations, including pressure sensitivity, vibration detection, and thermal perception [239]. Hydrogel-based sensors, with ionic and conductive properties, have demonstrated exceptional capabilities in overcoming these challenges [240]. For example, a biocompatible hydrogel tactile sensor with a hydrogel-ion channel configuration has been introduced, offering high sensitivity and rapid response to distinguish even minute pressure variations, making it suitable for advanced applications in prosthetic limbs and robotic touch [241]. Additionally, hydrogel-assisted electronic skins (e-skins) have been integrated into VR gloves, where strain sensors enable accurate hand tracking and electromechanical feedback that allows users to perceive resistance, surface textures, and dynamic tactile cues [242,243].

Hydrogel interfaces are also embedded in wearable haptic devices that significantly improve applications in telemedicine and remote robotic control. Hydrogel-integrated haptic gloves modulate hydration levels and ionic conductivity in response to user interactions, creating force feedback sensations [208,236]. For example, one study demonstrated the use of hydrogel-based gloves in VR surgical training simulations, where realistic touch responses enhanced immersion and control accuracy [244]. Moreover, a hydrogel-based neural interface has been developed to enable bidirectional communication between prosthetic limbs and the nervous system, facilitating near-natural touch perception and proprioception [245]. These interfaces, composed of conductive hydrogel layers, help minimize mechanical mismatch with biological tissues, ensuring long-term comfort and usability.

Hydrogel-based soft actuators have also contributed to innovations in haptic feedback systems by exhibiting dynamic responses to external stimuli such as electrical signals and temperature changes. These actuators enable real-time adaptation of tactile sensations [246]. For example, a hydrogel-based morphing interface simulates different surface textures within virtual environments to enhance realism and user engagement [247]. Hydrogel-based exoskeletons provide force feedback resistance, allowing users to experience sensations of hardness and weight in virtual objects, which is crucial for rehabilitation and industrial training [227,248].

Beyond mechanical feedback, hydrogel-based sensory materials integrated into haptic systems can deliver thermal and moisture-based feedback. Temperature-sensitive hydrogel composites, exhibiting thermochromic or phase change properties, simulate environmental changes in VR settings to improve immersive experiences [236]. For instance, a hydrogel-based thermal haptic pad generates localized temperature variations, replicating sensations such as warmth from a fire or coolness from an icy surface, significantly enhancing cognitive and emotional engagement in interactive and rehabilitation therapy applications [238,249,250].

Hydrogel interfaces are expected to become increasingly fundamental in advanced haptic systems, offering unparalleled realism and adaptability in HMI. Their soft, stretchable, and conductive nature makes them ideal for wearable haptics, prosthetics, and immersive digital environments, providing users with enriched sensory experiences [251]. Future research should focus on developing multifunctional hydrogel composites with improved self-healing properties and energy efficiency [252]. The integration of bioinspired hydrogel sensors with machine learning algorithms could enable personalized haptic feedback, further bridging the gap between virtual and physical interactions [253]. However, challenges such as limited mechanical durability, long-term stability, and dehydration-related performance degradation still hinder their widespread adoption, particularly in telemedicine and robotics [238]. Ongoing research efforts are crucial for overcoming these limitations, ultimately transforming how humans interact with advanced digital interfaces. Commercial products based on hydrogel interfaces in haptics and sensory systems are listed in Table 3.

## 7. Regulatory Requirements and the Patent Landscape of Hydrogel Biointerfaces

The regulatory requirements for hydrogel biointerfaces are contingent upon the specific components used in their formulation, making the regulations governing these hydrogel components directly applicable to the biointerfaces. Unlike drugs, which are broadly classified, hydrogels fall under the “devices” category according to Section 201(g) of the FD&C Act. In most cases, hydrogel-based products are required to undergo an additional FDA review through a 510(k) premarket notification submission to obtain legal marketing rights in the United States, a process that can take several years to complete. However, under the new European regulations, hydrogels are classified as Class III medical devices, requiring regulatory consideration throughout the entire lifecycle of the hydrogel—from material and machine qualification to scale-up. The Commission Regulation (EU) No. 722/2012, dated 8 August 2012, establishes specific requirements for active implantable medical devices and medical devices manufactured using animal tissues, addressing the need to maintain high levels of safety and health protection, particularly against the risk of transmitting animal spongiform encephalopathies [256]. This regulation mandates that class III active implantable medical devices undergo conformity assessment procedures before they can be placed on the market or put into service, including the adoption of more detailed risk analysis and management specifications. The regulation also outlines specific requirements for medical devices, including active implantable devices, made from animal-derived tissues such as collagen, gelatin, and tallow, which must meet at least the standards suitable for human consumption. Manufacturers or their authorized representatives are required to conduct risk analysis and risk management before applying for conformity assessment. Member states are responsible for verifying that bodies assessing medical device conformity have up-to-date knowledge and ensuring that only compliant devices are placed on the market or put into service. The conformity assessment for medical devices includes the evaluation of compliance with essential requirements under current directives and specific regulations. Manufacturers must collect, evaluate, and submit information on any changes to animal tissues or derivatives used in the devices and any related risk assessments. Two significant European Health Products Regulations came into effect on 26 May 2017. The first, Regulation (EU) 2017/745 on medical devices, which modifies Directive 2001/83/EC and repeals Council Directives 90/385/EEC and 93/42/EEC, has been applied since 26 May 2020. The second, Regulation (EU) 2017/746 on in vitro medical devices, replaces Directive 98/79/EC and Commission Decision 2010/227/EU and has been applied since 26 May 2022. These regulations mark a significant shift in the medical device sector, requiring stringent obligations for all market operators. This ensures enhanced transparency, traceability, and product safety [257]. As hydrogels are categorized as Class III medical devices under the new European regulations, this must be considered not only during scale-up, but also during the early stages of material and machine development. The regulations require that active implantable and Class III medical devices undergo conformity assessment procedures, incorporating more detailed risk management specifications. Additionally, provisions regarding the use of animal byproducts not intended for human consumption, as well as opinions on specified risk materials and minimizing the risk of animal spongiform encephalopathy transmission, should be considered. It is essential for the member states to ensure that the notified bodies have the necessary expertise and up-to-date knowledge to assess conformity effectively. The period for scrutiny granted to the competent authorities concerning the notified bodies should be carefully considered. The summary evaluation report for devices using certified materials should be shorter than for those using uncertified materials. The regulation also provides a transitional period for active implantable medical devices already covered by an EC design examination certificate or EC type examination certificate, allowing them to continue being placed on the market, in accordance with the opinions of the Committee on Medical Devices [258]. More details on regulatory aspects and individual hydrogel regulatory components are comprehensively summarized in other articles [256,257].

The rapid evolution of hydrogel-based biointerfaces has led to a surge in patent filings for advanced hydrogel formulations, smart hydrogels, and conductive hydrogel applications. Regulatory advancements have influenced how hydrogel patents are evaluated, particularly in terms of biocompatibility, long-term stability, and device integration. Table 4 lists the various hydrogel-based patents for HMI applications.

## 8. Challenges and Limitations of Hydrogel Interfaces in HMI

Hydrogels’ hydration-dependent properties, while beneficial for biocompatibility, pose challenges in real-world applications, particularly in maintaining durability and stability. One major issue is dehydration, as hydrogels lose water content in ambient or dry environments, reducing ionic conductivity and mechanical stiffening [268]. For instance, polyvinyl alcohol (PVA)-based hydrogels used in wearable sensors often require encapsulation to retain moisture, which can add bulk and compromise flexibility [269]. Recent advancements in hydrophobic–hydrogel composites, such as silicone–hydrogel hybrids, show promise in addressing this issue, but introduce fabrication complexities [205]. Additionally, hydrogels experience mechanical fatigue due to repeated stress, leading to microcracks or delamination over time. While stretchable hydrogels such as polyacrylamide–alginate networks can tolerate an over 500% strain, they suffer from hysteresis-induced signal drift. Self-healing hydrogels utilizing dynamic boronate ester bonds offer some recovery potential but struggle with slow healing kinetics under physiological conditions [270]. Another concern is in vivo degradation as enzymatic activity, pH fluctuations, and oxidative stress accelerate breakdown. PEG hydrogels, for example, degrade unpredictably in neural implants, causing electrode–tissue contact loss [271]. While strategies such as UV-induced cross-linking and enzyme-resistant monomers help improve stability, they also raise concerns about long-term biocompatibility. Beyond structural durability, signal consistency and power demands remain the key challenges in hydrogel-based sensors. Hydrogels have lower ionic conductivity compared to metals, which limits their sensitivity in high-fidelity applications such as neural recording. Incorporating conductive nanomaterials such as MXenes or graphene can enhance signal-to-noise ratios, but these modifications can increase impedance mismatches at the hydrogel–tissue interface [272]. Additionally, hydrogel sensors often have high power demands, especially in applications requiring continuous wireless monitoring. For instance, hydrogel-based glucose sensors necessitate frequent battery replacements, reducing patient compliance. Researchers are exploring energy-harvesting hydrogels, such as triboelectric nanogenerators, and low-power organic electrochemical transistors to address these limitations [273]. However, ion leaching and matrix swelling contribute to the baseline signal drift, posing challenges for long-term reliability, particularly in chronic neural interfaces where recalibration is impractical.

Scaling hydrogel-based devices from laboratory research to commercial products introduces additional hurdles. High production costs stem from the use of specialized monomers, cross-linking agents, and conductive nanomaterials. For example, carbon nanotube-reinforced hydrogels remain prohibitively expensive for large-scale use. While extrusion-based 3D printing reduces material waste, it struggles with achieving resolutions below 100 µm, limiting its applicability for fine-feature fabrication [274]. Sterilization methods also present obstacles as traditional techniques such as autoclaving or ethylene oxide treatment can degrade hydrogel networks. Although gamma irradiation has been effective for certain hydrogel formulations [275], it can alter the mechanical properties of synthetic polymers such as polyacrylamide. Aseptic manufacturing approaches, such as cleanroom 3D printing, offer a solution but remain cost-prohibitive for mass production. Regulatory approval further complicates commercialization, as agencies such as the FDA require extensive biocompatibility testing, especially for long-term implantable hydrogel devices. Furthermore, hydrogel batch-to-batch variability poses challenges in maintaining consistent product quality, which is essential for large-scale manufacturing.

Despite these challenges, progress has been made in enhancing the durability, signal reliability, and scalability of hydrogel-based sensors. Self-healing hydrogels and hybrid composites have improved structural resilience, although their in vivo healing kinetics require further optimization. Conductive hydrogel networks incorporating MXenes have enhanced signal consistency, but achieving impedance matching at biointerfaces remains a concern. Roll-to-roll 3D printing has facilitated scalable hydrogel production, yet batch variability still presents an obstacle [276]. Addressing these unresolved issues is crucial for advancing hydrogel-based HMIs, and ensuring their long-term functionality in wearable and implantable applications.

## 9. Future Perspectives

Future advancements in HMIs could be shaped by innovations in materials, biohybrid systems, and ethical considerations. The development of self-healing hydrogels using dynamic covalent chemistry may enhance durability by enabling autonomous repair of microdamage, which could improve longevity in wearable and implantable applications. Additionally, integrating hydrogels with advanced computational approaches, such as machine learning, might allow for real-time adaptation to physiological changes, improving sensor accuracy and responsiveness in biomedical and assistive technologies. Biohybrid systems incorporating living cells within hydrogels could enhance biocompatibility and functionality in applications such as tissue engineering, biosensing, and neural interfacing. These systems may facilitate better integration with biological tissues, enabling more effective interaction with medical implants or soft robotic systems [277]. Furthermore, advances in hydrogel conductivity and mechanical stability could improve their performance in flexible electronics, prosthetics, and neural prostheses, supporting the development of more responsive and adaptable biomedical devices. Moreover, with the increasing demand for sustainable materials, bio-derived components are playing a growing role in hydrogel-based biointerfaces. Plant-based polysaccharides, proteins, and lipids provide biodegradable and eco-friendly alternatives to conventional synthetic polymers. However, challenges remain in terms of mechanical properties, scalability, and consistency in performance. Addressing these limitations could further enhance the integration of bio-derived hydrogels, promoting both biocompatibility and sustainability. Future research could focus on optimizing their physicochemical properties and developing scalable fabrication methods for their application in wearable and implantable systems, as these technologies advance, ethical and societal considerations must be addressed. The increasing use of hydrogel-based interfaces in medical monitoring and neural interfacing raises concerns about data privacy and security, necessitating robust protection against unauthorized access and misuse. Additionally, ensuring equitable access to these technologies is important to prevent disparities in healthcare availability and human augmentation. Regulatory frameworks may need to evolve to address long-term biocompatibility, safety, and ethical concerns surrounding hydrogel-based biomedical and wearable systems. By addressing these challenges, hydrogel-based HMIs could contribute to the future of health care, rehabilitation, and human–machine interaction responsibly and sustainably.

## 10. Conclusions and Outlooks

Hydrogel-based wearable sensors have emerged as a key focus in human–machine interaction research due to their flexibility, biocompatibility, and responsiveness to physiological and environmental stimuli. While significant advancements have been made in enhancing the performance of hydrogel materials, integrating them effectively with artificial intelligence and smart HMI technologies remains a challenge. This review explored the design, fabrication, and functional characteristics of hydrogels in wearable HMI devices, emphasizing applications in healthcare monitoring, touch-sensitive interfaces, and intelligent system operations. Despite their potential, several challenges must be addressed for widespread adoption in HMI systems. One key issue is ensuring long-term stability and durability, as hydrogels lose water over time, affecting their mechanical properties and sensing performance. While hydrophilic additives such as glycerol help maintain hydration, they may alter polymerization dynamics and reduce sensor sensitivity. Additionally, integrating hydrogel sensors with electronic components requires optimization to achieve stable signal transmission and seamless functionality. Hydrogels’ inherently soft and hydrated nature can lead to compatibility issues when interfaced with rigid electronic components, necessitating innovative design strategies. Furthermore, there is a growing demand for miniaturized, cost-effective, and scalable hydrogel-based wearable sensors that can be used in diverse applications such as health care, robotics, and augmented reality. Achieving reliable performance in dynamic and unpredictable environments requires improving hydrogel adhesion, mechanical resilience, and signal accuracy. Addressing these challenges will enhance the role of hydrogels in next-generation HMI technologies, paving the way for more intuitive and efficient human–machine interactions.

## Figures and Tables

**Table 3:** Commercial hydrogel products for interfacing in haptics and sensory systems.

Product	Application	Hydrogel Interface Used	Reference
HapticGel VR Glove	Virtual reality (VR) haptic feedback	Conductive ionic hydrogel	[254]
Skinfeel e-skin	Wearable electronic skin (e-skin) for tactile sensing	Hydrogel-based electrode for neural signal transmission	[243]
Softsense prosthetic interface	Prosthetic limb sensory restoration	Biocompatible conductive hydrogel	[238]
Neurogel neural interface	Neural interfacing for sensory restoration	Hydrogel-based electrode for neural signal transmission	[255]

**Table 4:** Hydrogel-based patents for HMI applications.

S. No.	Patent No. and Country	Title	Intended Use	Details	Reference
1	US5854078A (USA)	Polymerized crystalline colloidal array sensor methods	Sensor devices	Hydrogels capable of shrinking and swelling in response to stimuli, changing light diffraction to detect concentration changes	[259]
2	US7794657B2 (USA)	Phase change sensor	Biosensors	Sensor material undergoes volume change upon target molecule binding, enabling signal detection	[260]
3	US20100272608A1 (USA)	Temperature sensor and biosensor using the same	Temperature sensors and biosensors	Evanescent wave excitation-based temperature and biomolecule detection	[261]
4	US8999378B2 (USA)	Porous electroactive hydrogels and uses thereof	Actuators and biomedical applications	Electroactive hydrogels with tunable deformation angle	[262]
5	US6835553B2 (USA)	Photometric glucose measurement system using a glucose-sensitive hydrogel	Glucose biosensor	Implantable biosensor using a hydrogel filament to detect glucose concentration photometrically	[263]
6	US20170151733A1 (USA)	Method of 4D printing a hydrogel composite structure	4D printing	Hydrogel composite structures with swelling-induced 3D shape transformation	[264]
7	US7482381B2 (USA)	Artificial muscle hydrogel blends reversibly electroactuated near neutral pH, implantable actuating devices, and methods using the same	Artificial muscles and drug delivery	Electroactuated hydrogel materials for fluid release and implantable actuators	[265]
8	US9084546B2 (USA)	Co-electrodeposited hydrogel-conducting polymer electrodes for biomedical applications	Biomedical electrodes	Bioelectrodes with enhanced biocompatibility for electronic signal detection	[266]
9	US8427433B2 (USA)	Tactile-feedback touchscreen	Tactile displays	Gel layer-based system providing tactile feedback for touchscreen interfaces	[267]

## Data Availability

Not Applicable.

## Abbreviations

The following abbreviations are used in this manuscript:

HMI	Human–machine interfacing
BMI	Brain–machine interfaces
CMC	Carboxymethyl cellulose
PVA	Polyvinyl alcohol
PAA	Polyacrylic acid
PAAm	Polyacrylamide
PEDOT	Poly(3,4-ethylenedioxythiophene)
PSS	Poly(styrene sulfonate)
PEDOT:SL-PAA	Poly(3,4-ethylenedioxythiophene): sulfonated lignin on poly (acrylic acid)
P(SBMA-co-AAm)	Poly(2-(methacryloyloxy) ethyl)dimethyl-(3-sulfopropyl)ammonium hydrox-ide-co-acrylamide)
LED	Light-emitting diode
ECM	Extracellular matrix
ECG	Electrocardiogram
EMG	Electromyogram
ECoG	Electrocorticography
AFP	Antifreeze proteins
FBR	Foreign body response
CNT	Carbon nanotube
LM	Liquid metal
LFP	Local field potential
GelMA	Gelatin methacrylate
GO	Graphene oxide
rGO	Reduced graphene oxide
PEG	Polyethylene glycol
IOP	Intraocular pressure
PAc	Polyactylene
PTh	Polythiophene
